# Fungi Classification in Various Growth Stages Using Shortwave Infrared (SWIR) Spectroscopy and Machine Learning

**DOI:** 10.3390/jof8090978

**Published:** 2022-09-19

**Authors:** Zhuo Liu, Yanjie Li

**Affiliations:** Research Institute of Subtropical Forestry, Chinese Academy of Forestry, Hangzhou 311400, China

**Keywords:** dark septate endophytes (DSEs), fungi identification, preprocessing, support vector machine (SVM), variable selection

## Abstract

Dark septate endophytes (DSEs) fungi are beneficial to host plants with regard to abiotic stress. Here, we examined the capability of SWIR spectroscopy to classify fungus types and detected the growth stages of DSEs fungi in a timely, non-destructive and time-saving manner. The SWIR spectral data of five DSEs fungi in six growth stages were collected, and three pre-processing methods and sensitivity analysis (SA) variable selection methods were performed using a machine learning model. The results showed that the De-trending + first Derivative (DET_FST) processing spectra combined with the support vector machine (SVM) model yielded the best classification accuracy for fungi classification at different growth stages and growth stage detection on different fungus types. The mean accuracy of generic model for fungi classification and growth stage detection are 0.92 and 0.99 on the calibration set, respectively. Seven important bands, 1164, 1456, 2081, 2272, 2278, 2448 and 2481 nm, were found to be related to the SVM fungi classification. This study provides a rapid and efficient method for the classification of fungi in different growth stages and the detection of fungi growth stage of various types of fungi and could serve as a tool for fungi study.

## 1. Introduction

Endophytes fungi, which mainly inhabit healthy plant tissues, have been widely noticed due to their diversity and ecological significance in recent years [1]. It is a crucial component in plant root mycobiome and could reveal masked, undiscovered fungal diversity [2]. Dark septate endophytes (DSEs) are able to alleviate the host plant abiotic stresses [3] by producing melanized hyphae and microsclerotia in host plant roots which are crucial for host salt tolerance [4]. However, different DSE species could yield various influences on their host plant for abiotic stresses [5]. As a result, it is of vital importance to study the growth characteristics of DSEs and identify various DSEs species, so as to understand the influence of fungal growth on host plants.

Numerous studies have been carried out on fungal identification and growth characteristics study using traditional techniques, including enzyme assays, time lapse cameras, cytochemical processing and photo microscopy [6], which are cost- and time-consuming and often lead to further biological or chemical contamination [7], and are not suitable for high-throughput and rapid measurement. Therefore, a non-destructive and highly efficient method is needed.

Near-infrared (NIR) spectroscopy, a vibrational spectroscopy ranged 800–2500 nm, mainly used in hydrogen-containing groups such as C–H, O–H, N–H and S–H stretching modes, has received extensive attention for its great potential in many fields [8,9,10,11]. In particular, NIR has been shown to have advantages in the classification of fungi species [12] and monitoring fungi growth stages [13]. To create a robust and promising classification model, spectral pre-processing and important variable selections need to be coupled with pattern recognition algorithms, such as support vector machine (SVM) [14], random forests (RF) [15] and multilayer perceptron (MLP) [16]. SVM, a data-driven approach for pattern recognition tasks, has extended its usage from initially binary classification to multiclass classification, which allows for a broad range of classification approaches in spectroscopy and remote sensing [17,18]. RF is also a powerful algorithm that could be used for solving both classification and regression problems [19]. It was first introduced by Breiman [15] and has been used in a broad field, including ecology [20], agriculture [21], and fungi study [22,23]. MLP is a deep learning methodology [24], widely used for classification and regression in recent years [25,26]. It mainly contains three types of layers: the input layer, the output layer, and the hidden layer. The input layer will load the input data to be processed, the output layer needs to be set based on the task of prediction or classification, and the true computational engine of the MLP is the multiple hidden layers which locate between the input and output layer. Pattern classification, recognition, prediction, and approximation are the main uses of MLP [27].

Spectral pre-processing methods are mainly used to reduce the overlapping and noise influence from the raw NIR spectra which may reduce the model accuracy. The most common spectral pre-processing methods are block scaling (BS) [28], De-trending (DET) [28], and first (FST) and second (SED) derivatives [29]. Spectra not only contain the useful information that could highly contribute to the model accuracy, but also contain irrelevant variables which may reduce the model accuracy [30]. Therefore, it is crucial to use the most important variable that is highly correlated with the target traits instead of the full spectra band. There are many variable selection algorithms available including genetic algorithm (Ga) [31], Sub-window permutation analysis (SwPA) [32], backward variable elimination (BVE) [33], significant Multivariate Correlation (sMC) algorithm [34], and sensitivity analysis (SA) [35]. Among them, the Ga, SwPA, BE, and sMC methods are mostly applied on the partial least-squares regression (PLSR) algorithm [36,37,38]. The SA techniques have been studied for the selection of important variables in recent years [39,40,41]. The SA method usually considers the effects of both individual input variables and the interaction among input variables on the output, and it works well on the SVM model for classification [42].

Although it has been proved that the NIR spectroscopy can be used for fungi detection, limited research outcome has been found on its use in the classification of different fungal species in a variety growth stages [12,43]. Lu, Wang, Huang, Ni, Chu, and Li [12] evaluated and classified five cereal fungi in different growth stages using Visible/Near-Infrared hyperspectral imaging and machine learning algorithm ranged from 400 to 1000 nm and yielded a satisfactory result using SVM model coupled with the successive projection algorithm (SPA) wavelength selection method. However, to the best of our knowledge, there is no research that identifies endophytes fungi on the culture medium in different growth stages using the shortwave NIR spectroscopy (SWIR). SWIR ranges from 1100–2500 nm, which is relatively low compared to the visible and near-infrared region (VNIR, 400–1100 nm), mainly due to its lack of sufficient technology and high-cost silicon detectors [44]. However, compared to the VNIR region, SWIR has the advantages of easy penetrating the atmosphere, sensitive to the water content, high total transmittance in the atmospheric, and containing many unique absorption features that are not available in the VNIR [45].

To fill the gap, the study aims to use the SWIR technique combined with machine learning methodology to evaluate the possibility on the classification of different fungi in different growth stages, the specific objectives are: (1) comparing the advantages of different machine learning models to establish an optimal model for fungi species classification; (2) exploring the possibility of classifying different endophytes fungi in an early stage and determining the earliest classifiable time; (3) selecting the most relevant wavelengths correlated with the classification of different endophytes fungi and different growth stages on model building; (4) testing the possibility of using a mixed growth stage model with optimal informative variables for a rapid fungi classification.

## 2. Materials and Methods

### 2.1. Sample Preparation

**Figure 1:**
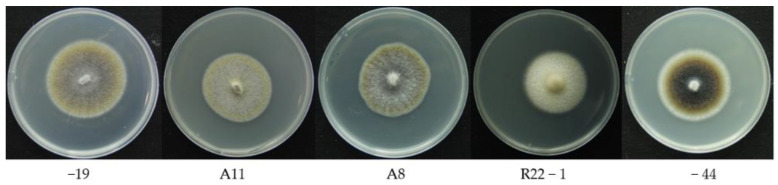
Five strains of *Laburnicola rhizohalophila* in the same growth stage.

Five strains of *Laburnicola rhizohalophila* (Figure 1) [46] purchased from the China General Microbiological Culture Collection Center (CGMCC 3.19615) were propagated on the modified Melin-Norkrans liquid medium [47] at 28 °C for 7 days to obtain the pure fungi with high vigor and consistent activity. Then, the fungi were inoculated on potato dextrose agar (PDA) and placed in a mold incubator at 22 °C, with 22 identical replicates of each group. A total of 110 spectra were obtained for each growth stage of five fungi, and a total of 660 spectra were obtained for five species.

### 2.2. SWIR Spectrum Acquisition

To monitor the growth stages of each fungi, the SWIR spectra were collected every two days with an interval of 1 h from the first day after inoculation. The NIR spectra data were taken from the top of the Petri dish with a lid using the field-based spectrometer (LF-2500, Spectral evolution, Haverhill, MA, USA) with a handheld fiber optic contact probe. Each spectrum was averaged 24 scans and saved in a 6 nm resolution with a range of 1100 to 2500 nm.

### 2.3. Model Calibration and Validation

SVM, RF, and MLP, three common machine learning methods, were generated to obtain the optimal classification model for five endophytes fungi. Three different spectral pre-processing methods and their combination (BS, DET, FST, DET_BS, DET_FST) were performed on the spectrum of each sample when building classification model to reduce the effects of overlapping or light noise at different wave numbers [48]. A variable selection method called sensitivity analysis (SA) [49] was applied to extract the most relevant spectra variables from the pre-processing or raw spectra that contribute highly to the accuracy of the classification model. Data were randomly split into three sets: calibration set (60%), validation set (20%), and test set (20%). To check the stability of overall data on the classification models, each model with different pre-processing methods and variable selection methods were repeated 100 times.

### 2.4. Model Evaluation

To check the quality of the calibration models, four parameters, i.e., overall accuracy (OA), precision (P), recall (R), and the confusion matrix of each model were generated. Similar to the study of Li, et al. [50], we used true positives (TP), false positives (FP), true negatives (TN), and false negatives (FN) calculated from the confusion matrix of each model. Since the models in this study are for multiclass classification, the confusion matrix will have a N × N dimension where N is the number of different class labels C0, C1, …, CN (e.g., N = 5 for fungi in this study). The performance metrics for the OA and the specific class $C_{i}$ in the model can be expressed as follows [51]:

(1) $$\mathrm{OA}=\frac{\sum \begin{array}{c} N\\ i=1 \end{array}\;\mathrm{TP}\left(C_{i}\right)}{\sum \begin{array}{c} N\\ i=1 \end{array}\;\sum \begin{array}{c} N\\ j=1 \end{array}C_{i,j}\;}$$

(2) $$R=\frac{\mathrm{TP}\left(C_{i}\right)}{\mathrm{TP}\left(C_{i}\right)+\mathrm{FN}\left(C_{i}\right)}\;$$

(3) $$P=\frac{\mathrm{TP}\left(C_{i}\right)}{\mathrm{TP}\left(C_{i}\right)+\mathrm{FP}\left(C_{i}\right)}\;$$

All of the analyses were conducted using R software [52] integrated with R studio [53]. The *caret* [54] packages have been used for SVM modeling, the *prospectr* [55] package for spectra pre-processing, the *rminer* package [56] for variable selection, and the *ggplot2* [57] for data visualization.

## 3. Results

### 3.1. Fungi Classification in Different Growth Stages

**Figure 2:**
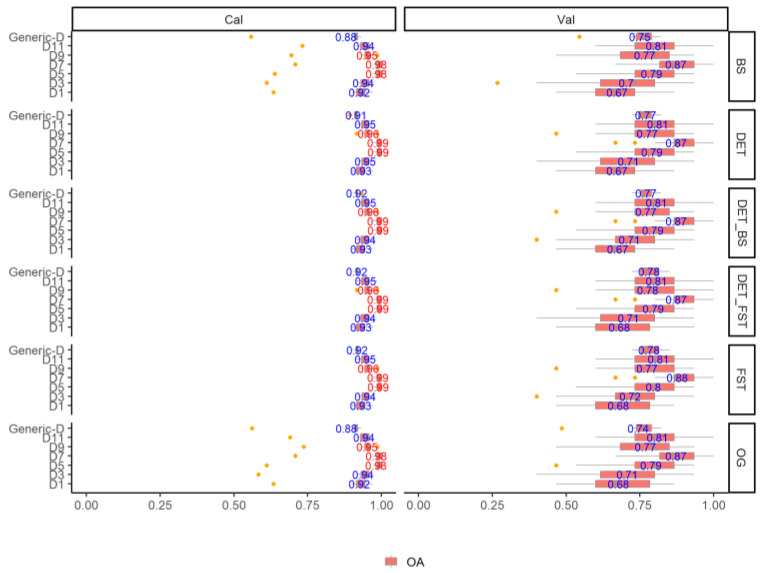
The overall accuracy (OA) Distribution (95% confidence intervals) of calibration and validation statistics from 100 simulations of models classifying the fungi in different growth stages with different spectra pre-processing methods using the SVM model.

**Figure 3:**
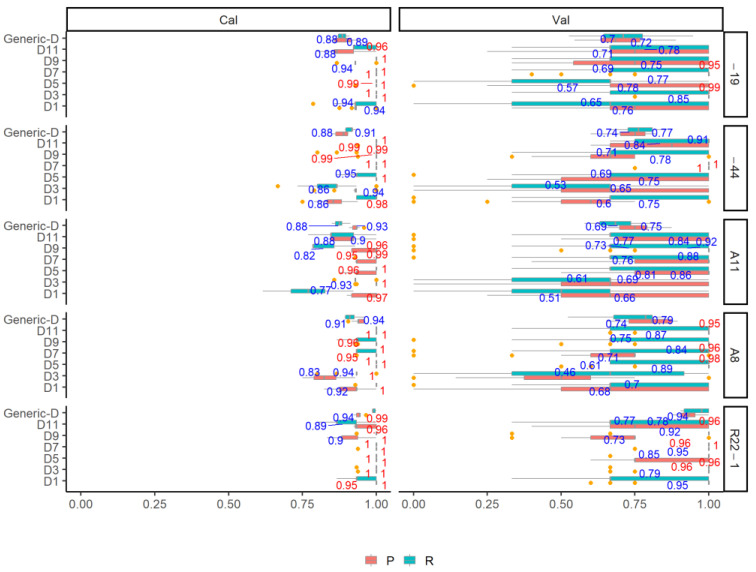
The performance of P and R in different fungi using the optimal DET_FST pre-processing methods and SVM model in different growth stages. Values more than 0.95 are shown in red, otherwise in grey.

Figure 2 shows the OA results of fungi classification using the SWIR spectra in six individual growth stages and generic model (generic-D). Regardless of the pre-processing methods, all the individual growth stage models and the generic model yield a promising and reliable classification accuracy with the mean OA_Cal_ higher than 0.9. The DET_FST and FST pre-processing methods yield the highest mean OA_Cal_ with a range from 0.92 to 0.99 in all of the models, followed by the DET_BS (range: 0.92–0.99) and DET (range: 0.91–0.99), the BS pre-processing and the original spectra yield the lowest mean OA_Cal_ (range: 0.92–0.98). When using the DET_FST processing spectra for model classification, different growth stages influence the OA_Cal_, with the highest mean OA_Cal_ of 0.99 in D5 and D7. D1 yields the lowest classification result compared to other individual growth stage models, but still high with the mean OA_Cal_ of 0.93. The generic-D model using of all growth stage data yield a satisfactory classification result with the highest mean OA_Cal_ of 0.92. The classification error is low in calibration and slightly high in validation from the 100 simulated models for both generic and individual growth stages. The P and R of the calibration and validation set using the DET_FST processing spectra in all models are shown in Figure 3. All of the fungi show a stable P_Cal_ and R_Cal_ in all different growth stages, The highest mean P_Cal_ and R_Cal_ (range: 0.89–1) is found in R22-1 fungi, and A11 shows the lowest mean P_Cal_ and R_Cal_ (range: 0.77–1) compared to other fungi.

### 3.2. Early Detection of Fungi in Different Species

**Figure 4:**
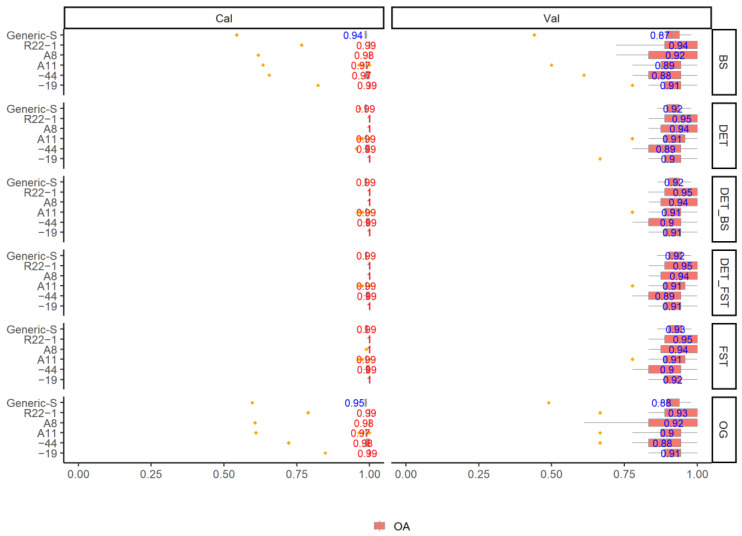
The overall accuracy (OA) Distribution (95% confidence intervals) of calibration and validation statistics from 100 simulations of models classifying the fungi growth stages in different types of fungi with different spectra pre-processing methods using the SVM model.

**Figure 5:**
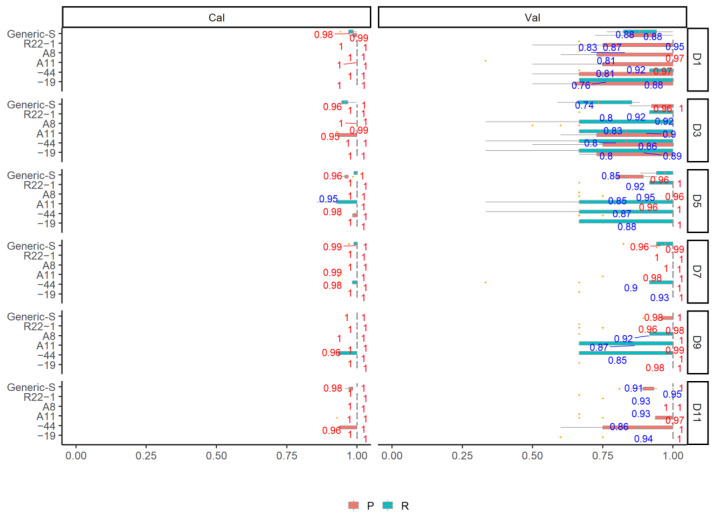
The performance of P and R in different growth stage using the optimal DET_FST pre-processing methods and SVM model on different types of fungi. Values more than 0.95 are shown in red, otherwise in grey.

The results of classification on different growth stages using five fungi are shown in Figure 4 and Figure 5. It can be clearly seen that the growth stages of different fungi have been successfully detected using various pre-processing methods. All of the models yield a high mean OA_Cal_ value ranging from 0.97 to 1. Pre-processing methods have slightly improved the model accuracy. DET, DET_BS, DET_FST, and FST yield similar mean OA_Cal_ (range: 0.99–1) in all individual sample model and generic model. Models using BS processing spectra and original spectra without processing yield promising classification OA_Cal_, but with some OA_Cal_ outliers from the 100 simulated model. Similarly, when choosing the DET_FST processing spectra to generate models, the P and R are high in both calibration and validation set in different types of fungi. All of the P _Cal_ and R _Cal_ in six growth stages are higher than 0.95. In addition, the generic model (Generic-S) for detecting the different growth stages using all types of fungi yield a high and promising P _Cal_ and R _Cal_ result for all growth stages, with P _Cal_ and R _Cal_ mean value ranging from 0.96 to 1. The data show a stable result with small error in OA, P _Cal_ and R _Cal_ for 100 simulated calibration models.

### 3.3. Model Comparison

After selecting the best pre-processing method using SVM model, other machine learning methodologies were also applied and compared to the SVM model using DET_FST processing spectra. The OA results show that other machine learning models also provide a stable and promising result in both fungi classification and early detection (Table 1). The OA value of RF_Cal_ and MLP_Cal_ are 0.91 and 0.91 in fungi classification and 0.99 and 0.98 in early detection, respectively.

### 3.4. Model Evaluation for Fungi Classification

**Figure 6:**
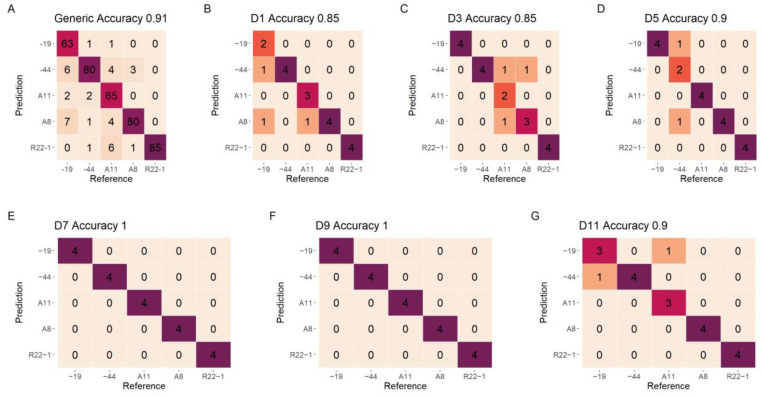
The confusion matrixes of fungi classification for test data of individual growth stages and the generic growth stages model using DET_FST processing spectra. (**A**) The generic model; (**B**) D1 model; (**C**) D3 model; (**D**) D5 model; (**E**) D7 model; (**F**) D9 model; (**G**) D11 model. The color changed from yellow to fuchsia represent different classified numbers.

The confusion matrixes for fungi classification of various growth stages and the generic model on the test data set are plotted in Figure 6. The model using the D1 and D3 data shows the lowest result on test data, with only 0.85 accuracy. As the fungi grow, the classification accuracy shows a growing trend, with 1 classification accuracy on the D7 and D9, respectively. The generic model using all growth stages data show slightly low classification accuracy on the test set (0.91). The most unstable fungi for classification is −19, which has misclassified six samples to −44, two samples to A11, and seven samples to A8.

### 3.5. Model Evaluation for Early-Stage Detection

**Figure 7:**
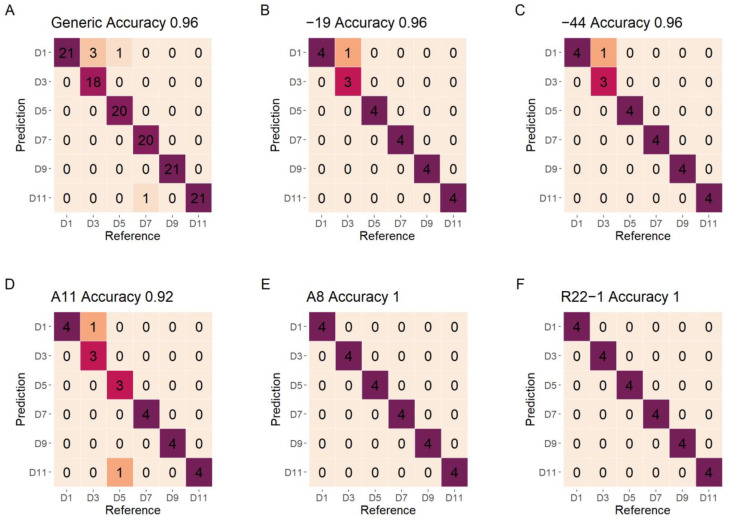
The confusion matrixes of growth stages detection for test data of individual fungi and the generic fungi model using DET_FST processing spectra. (**A**) The generic model; (**B**) −19 model; (**C**) −44 model; (**D**) A11 model; (**E**) A8 model; (**F**) R22−1 model. The color changed from yellow to fuchsia represent the different classified numbers.

The confusion matrixes for the classification of different growth stages using various fungi and generic fungi models on the test set are shown in Figure 7. All of the models show a high classification result with accuracy higher than 0.92. Different types of fungi show a slight difference on the detection of growth stages, The R22−1 and A8 fungi show an accuracy of 1, followed by the −44 and −19 fungi (accuracy of 0.96). The A11 fungi show the lowest classification accuracy of 0.92 on test data. The generic model for different growth stages shows a high and reliable accuracy on the test data (0.96).

### 3.6. Importance Variable Selection

**Figure 8:**
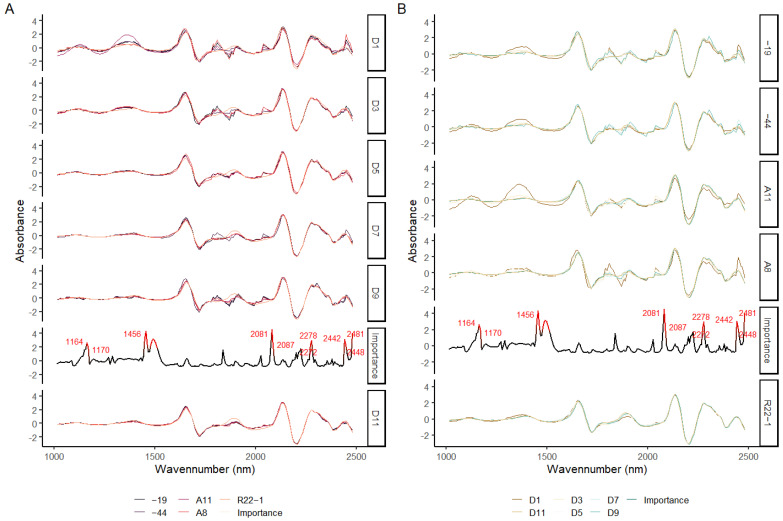
The DET_FST processing spectra of different fungus types (**A**) and growth stage (**B**); the importance values of wavelengths for fungi classification (**A**) and growth stage (**B**) detection using SVM model and DET_FST processing spectra.

The average of DET_FST processing spectra for five fungi and six growth stages and the important variables for these two kinds of SVM models are shown in Figure 8. For the averaged spectra of fungi samples, the region around 1800 to 2000 nm and 1300 nm are found to have various changes for different fungi types with growth (Figure 8A), and similar results are also found in the growth date of different fungi (Figure 8B). The top 10% important spectral variables are labeled in red. Similar important variables are found for both fungi classification model and growth stage model, which are 1164, 1456, 2081, 2272, 2278, 2442, 2448, and 2481 nm, respectively. The important variables found by the SA from SVM model are located slightly differently to the change area of DET_FST processing spectra.

## 4. Discussion

The SWIR technology, in our study, has been successfully applied to the DSEs fungi classification and the early growth stage detection. It shows a promising and reliable result using machine learning methodology. To the best of our knowledge, it is the first report on the DSEs fungi classification using SWIR spectra. Similar study on fungi classification and growth stage detection using NIR spectra was made by Lu, Wang, Huang, Ni, Chu, and Li [12], who reported that the cereal fungi can be successfully classified in all growth stages using the SVM model and successive projection algorithm variable selection methods combined with the Visible/Near-Infrared (Vis/NIR) hyperspectral imaging, with an overall accuracy of more than 95.87% on all growth stages and 98.89% on the first growth stage, which is close to our study (with a mean accuracy of 0.92 on all growth stages and 0.99 on the first growth day). In addition, SWIR hyperspectral imaging has also shown a promising ability on the *Fusarium* spp. Fungi detection using the partial least squares discriminant analysis (PLS-DA) models yielded a reasonable accuracy ranging from 78% to 100% on the train and test data sets [58]. Our results showed that different growth stages influence the fungi classification, with an OA value ranging from 0.92 to 0.99, supported by the study of Sun, Gu, Wang, Huang, Wei, Zhang, Tu, and Pan [13], who found that the best accuracy in the classification of three spoilage fungi is 97.5% for the test dataset at 36 h using the 400 to 1000 nm NIR hyperspectral imaging and PLSDA model.

Three spectra pre-processing methods (BS, DET, FST) and their combination (DET_BS, DET_FST) were applied in our study to check the influence of processing spectra on the SVM classification accuracy. Our results found that BS showed no improvement on the model accuracy compared to the original raw spectra with more outliers when simulated 100 times, and that DET, DET_BS, DET_FST showed high improvement on both fungi classification and early detection models, with the highest OA reaching 1. Our results are different to what were reported in the study by Wu, et al. [59] who found the highest classification accuracy of waxy wheats was gained by using the raw non-processing SWIR spectra data combined with the SVM model, reaching 98.51%. Different applications may lead to different results. Xu, et al. [60] found the second-order derivatives and standard normal variate (SNV) processing spectra can largely enhance the classification of regular and aged shiitake using SVM model. Yao, et al. [61] found that the combination of different pre-processing methods (second derivative (2D), Savitzky–Golay (SG) filter and SNV) could improve the SVM model accuracy on the discrimination of five Boletaceae mushroom species, which is similar to our study where the combination of DET and first derivative yield the optimal classification results on the SVM model. Therefore, the spectral preprocessing methods may need to be performed based on the needs of practical applications [62].

Different types of machine learning methodologies including SVM, RF, and MLP have been compared in our study to find the most suitable model for fungi classification and growth stage detection. It has shown that all of the machine learning methodologies can achieve high classification aspects. All of the classification accuracy on the calibration set for the two kinds of models are above 0.9. Though all these three machine learning models can be applied to the fungi classification and growth stage detection, since the SVM model performed slightly better than the other two methods, we selected the SVM as the final model in our study, which is supported by Castro, et al. [63] who compared three classifiers, decision tree (DT), SVM, and K-nearest neighbor, and found that the SVM model yielded the best performance on the classification of different growth stages of coffee rust infection, using the Vis/NIR (400–1000 nm) hyperspectral imaging. In addition, the detection and classification of corn (*Zea mays* L.) seeds also yielded a high classification accuracy of 96.46% when SVM was applied using SWIR hyperspectral imaging [64].

The region of SWIR (1100–2500 nm) has been well studied for whether it is highly sensitive to the target water, lignin, cellulose, and proteins [13,65]. Seven prominent bands around 1164, 1456, 2081, 2272, 2278, 2448, and 2481 nm were found by SA variable selection method in our study which has highly contributed to the SVM classification model. These bands found to be related to the model classification are not exactly equal to the findings of other studies, maybe because the different spectral processing varied the number of bands in the spectral range [11]. However, similar bands can be found by other studies. The bands around 1164, 1456, and 2081 nm have been reported to be highly related to the carbohydrates [6,66], the bands at 2272, 2278 are mostly corelated to the stretching or deformation vibration of C-H or C-C, and the bands at 2448 and 2481 nm have been reported to be related to protein contents [11,67]. All of these band regions were responsible for the differences between fungi species and growth stages.

## 5. Conclusions

In our study, the growth stages of five different DSEs fungi inoculated on the PDA were classified using the SWIR spectroscopy based on machine learning models. It has been shown that the SWIR spectroscopy can be successfully used for the fungi classification in different growth stages and the growth stage detection in different fungi. Generic model for the two approaches also provides a reliable and promising classification accuracy. Furthermore, the hyperspectral imaging with SWIR regions should be studied in future to yield a better result for visualization. Our classification of fungi and detection of fungi growth stages using the SWIR spectroscopy is fast and accurate which could enhance the fungi study in the future.

## Figures and Tables

**Table 1:** The mean OA accuracy of 100 simulated generic models for the fungi classification and early detection using SVM, RF, and MLP methodology and the optimal DET_FST processing spectra.

Model Types	Generic Model	OA	
		Cal	Val
Fungi classification	SVM	0.92	0.76
	RF	0.91	0.75
	MLP	0.91	0.77
Early detection	SVM	0.99	0.92
	RF	0.99	0.92
	MLP	0.98	0.91

## Data Availability

The data mentioned in this paper are available on request from the corresponding author.
